# Reply to S Rahman and S Ireen

**DOI:** 10.1093/ajcn/nqz055

**Published:** 2019-05-16

**Authors:** Christine P Stewart, Kathryn G Dewey, John M Colford, Mahbub Rahman, Stephen P Luby

**Affiliations:** 1 From the Department of Nutrition, University of California, Davis, Davis, CA; 2 Division of Epidemiology and Biostatistics, University of California, Berkeley, Berkeley, CA; 3 International Center for Diarrheal Disease Research, Dhaka, Bangladesh; 4 Division of Infectious Diseases and Geographic Medicine, Stanford University, Stanford, CA

Dear Editor:

We thank Rahman and Ireen for their interest in our recent publication (1). Indeed, we had been surprised to find that the prevalence of anemia was lower than expected in this study site in Bangladesh (2). The control group prevalence in our study was 17.4%, just more than half the 33% prevalence reported in the National Micronutrient Status Survey published in 2013 (3) and substantially lower than the 48.8% prevalence that we found in Kenya (2). We agree with the sentiment in the letter that groundwater iron concentrations are an important contributor to population iron status in Bangladesh, but we are not convinced that this is the reason for the unexpectedly low prevalence of anemia in our study compared with that in other areas of Bangladesh. Because it was shown previously that iron status is correlated with groundwater iron concentration (4), we had purposefully selected an area with low groundwater iron concentrations (5). According to the Bangladesh National Hydrochemical Survey, the majority of groundwater iron concentrations should have been <2 mg/L in our study area, which we illustrated in Supplemental Figure 1. Indeed, median groundwater iron concentration measured in the study area prior to the start of the intervention trial was 0.91 mg/L (IQR: 0.36–2.01 mg/L) (6). Iron deficiency did appear to be a problem in our study area. The prevalence of iron deficiency was 41% (inflammation corrected ferritin <12 µg/L or serum soluble transferrin receptor >8.3 mg/L), which was reduced by 40–60% in the 2 nutrition intervention groups. We have struggled to explain the unexpectedly low prevalence of anemia in this area compared with that of the national survey (3), which included sampling from regions that had much higher groundwater iron concentrations. One possibility that we had considered was that our blood sampling methods differed from those used in the survey, which we erroneously stated had used capillary blood sampling. However, Rahman and Ireen have correctly noted that we did in fact use the same method of venous blood sampling. Nevertheless, it is apparent from our study as well as from the national survey that micronutrient deficiencies are a problem in Bangladesh, regardless of the prevalence of anemia, and that the prevalence likely varies regionally. We recommend that investigators measure groundwater iron concentrations in future studies of iron or other micronutrient interventions.
